# Revolutionizing nanosatellites’ data integrity with SEEnet: A real-time ensemble learning approach for Single-Event Effect (SEE) prediction

**DOI:** 10.1371/journal.pone.0347344

**Published:** 2026-04-30

**Authors:** Sara Karim, Ekramul Haque Tusher, Abdur Rahman, Riadul Islam Rabbi, K. O. Anwar, Khair Razlan Othman

**Affiliations:** 1 Department of Space System Engineering, Aviation and Aerospace University, Dhaka, Bangladesh; 2 Faculty of Computing, University Malaysia Pahang Al-Sultan Abdullah, Pahang, Malaysia; 3 Department of Electrical Engineering and Automation, Harbin Institute of Technology, Shenzhen, China; 4 Faculty of Engineering and Technology, Multimedia University, Melaka, Malaysia; 5 Centre for Advanced Analytics, COE for Artificial Intelligence, Faculty of Engineering and Technology (FET), Multimedia University, Melaka, Malaysia; Instituto Nacional de Pesquisas Espaciais, BRAZIL

## Abstract

As nanosatellites make access to space more affordable and widespread, protecting onboard data from radiation-related damage has become a major challenge for modern low-cost missions. These small satellites often rely on commercial off-the-shelf (COTS) electronic components, which are particularly vulnerable to radiation-induced Single-Event Effects (SEEs) that can disrupt system operation and compromise mission data integrity. To address this challenge, our study proposes SEEnet, a lightweight ensemble learning framework designed for real-time prediction of SEE occurrence in nanosatellite systems. SEEnet combines multiple decision-tree classifiers with varying model depths using a soft-voting strategy, allowing it to improve prediction reliability while maintaining low computational complexity suitable for resource-constrained onboard environments. Our proposed approach is evaluated using a publicly available dataset from the Institute of Space Systems, University of Stuttgart, describing satellite spatial characteristics and associated SEE events. Experimental results show that SEEnet achieves a classification accuracy of 77%, outperforming several baseline machine-learning models, including Support Vector Machines, Random Forests, and Gradient Boosting, under the same evaluation conditions. In addition, the model demonstrates balanced precision–recall performance and provides bootstrap-based uncertainty estimates, enhancing confidence in its predictions. Overall, the results indicate that SEEnet offers an effective and computationally efficient solution for early SEE risk assessment, supporting proactive fault mitigation and improved data reliability in real-time nanosatellite missions.

## Introduction

Nanosatellites, commonly referred to as CubeSats, have emerged as transformative platforms in space exploration, communication, scientific research, and Earth observation due to their compact size, cost efficiency, and operational flexibility [1]. These advantages have significantly lowered the barriers to space access, enabling broader participation by universities, startups, and independent research groups [2]. As a result, the number of nanosatellite missions has increased rapidly over the past decade, reflecting their growing importance in modern space applications.

Despite their expanding role, nanosatellites face substantial operational challenges, particularly in maintaining data integrity within harsh space environments [3]. These environments are characterized by elevated radiation levels, extreme thermal variations, and continuous exposure to high-energy particles such as cosmic rays and solar energetic particles [4]. Among the various radiation-induced phenomena, Single-Event Effects (SEEs) pose a critical threat to nanosatellite reliability and mission success [5]. SEEs occur when energetic particles strike sensitive regions of semiconductor devices, causing unintended changes in circuit behavior that may manifest as transient faults, memory bit flips, or more severe system-level disruptions. Historical incidents highlight the practical consequences of such effects. For example, in April 2023, Beidou Navigation Satellites experienced memory bit flips induced by heavy-ion radiation, leading to data corruption and temporary service degradation [6]. Similarly, a solar particle event in November 2000 caused significant noise in the SOHO/LASCO imager, an effect attributed to SEE-related radiation impacts that required corrective action [7].

Global launch statistics further emphasize the urgency of addressing these challenges. From 1999 through May 2024, more than 2,600 nanosatellites have been launched worldwide, with a sharp increase observed over the last decade [8]. Notably, the successful deployment of CubeSats beyond Low Earth Orbit (LEO) and the integration of onboard propulsion systems demonstrate both technological maturity and expanding mission complexity. As nanosatellite missions grow in duration, autonomy, and operational scope, ensuring robust protection against radiation-induced failures becomes increasingly critical.

Nanosatellites are particularly susceptible to radiation effects because they frequently rely on Commercial Off-The-Shelf (COTS) electronic components. While these components offer cost and availability advantages, they are not inherently designed to withstand the radiation-rich conditions of space [9]. Consequently, even minor radiation-induced errors can compromise scientific measurements, disrupt communication links, or lead to partial mission failure. Traditional mitigation strategies such as radiation shielding or hardware redundancy are often impractical for nanosatellites due to strict constraints on mass, power consumption, and cost. This limitation has motivated growing interest in software-based and data-driven mitigation approaches.

In this context, machine learning provides a promising alternative for proactive SEE risk assessment. By analyzing telemetry and environmental data, machine learning models can estimate the likelihood of radiation-induced anomalies before they result in critical failures [10–13]. However, many existing approaches rely on computationally intensive models, offline analysis, or post-event fault detection. Deep learning techniques, while powerful, often demand substantial processing resources and lack interpretability, making them less suitable for real-time onboard deployment on resource-constrained nanosatellite platforms. Furthermore, several prior studies focus on isolated SEE types or lack integration with spatial and environmental context, limiting their operational applicability.

The primary motivation of this research stems from the growing reliance on nanosatellites for cost-effective space missions and their increased vulnerability to radiation-induced failures due to the use of commercial off-the-shelf electronic components. Traditional mitigation strategies, such as radiation hardening and hardware redundancy, are often impractical for nanosatellites because of strict constraints on mass, power consumption, and cost. At the same time, while recent research has explored radiation effects using physical modeling and hardware-based solutions, comparatively fewer studies have focused on lightweight, computational approaches capable of providing early warning and predictive mitigation in real time. This creates a clear need for software-based solutions that can operate onboard using readily available telemetry data.

Motivated by these limitations, this paper proposes SEEnet, a lightweight ensemble-based machine learning framework for real-time prediction of SEE occurrence in nanosatellite systems. SEEnet leverages an ensemble of optimized decision-tree classifiers combined through a probabilistic soft-voting mechanism, enabling robust prediction performance while maintaining low computational overhead. The framework incorporates bootstrap-based uncertainty estimation to enhance prediction reliability, particularly under class-imbalanced conditions. By prioritizing efficiency, interpretability, and real-time feasibility, SEEnet is designed to support early warning and mitigation strategies such as memory scrubbing or adaptive operational control.

The main contributions of this study are summarized as follows:

1. An analysis of the limitations of existing SEE mitigation and prediction techniques in the context of nanosatellite missions.
2. The development of SEEnet, a lightweight ensemble learning framework tailored for real-time SEE prediction using spatial telemetry and environmental context.
3. A comprehensive evaluation of SEEnet against commonly used machine learning models, demonstrating its effectiveness and suitability for integration into real-world nanosatellite platforms.

## Related work

The increasing prevalence of nanosatellites in space missions has raised concerns about the impact of space environments on the integrity of their onboard systems, particularly regarding Single-Event Effects (SEEs). The adverse impact of cosmic radiation, solar winds, and high-energy particles on satellite electronics has been widely studied.

Hillier et al [14] provided a comprehensive review of Error Detection and Correction (EDAC) systems specifically designed for nanosatellites. Their paper emphasized the unique challenges that space radiation poses to these small satellites and highlights the importance of maintaining data integrity. They evaluated various EDAC techniques, including Hamming codes and cyclic redundancy checks (CRC), which are critical in mitigating Single-Event Effects (SEEs). The review also discussed recent advancements in EDAC technology, underscoring its growing importance in modern nanosatellite missions. The authors concluded that the adoption of advanced EDAC techniques is essential for ensuring reliable performance in space environments, where radiation-related errors can severely compromise mission success.

Ferrer et al [15] focused on Medium Access Control (MAC) protocols used in satellite-based Internet of Things (IoT) systems, with a particular emphasis on nanosatellites. The authors review and evaluate the performance of various MAC protocols, highlighting the potential of CubeSats to provide cost-effective and versatile solutions for IoT connectivity. They address challenges such as latency, energy efficiency, and scalability, which are crucial for ensuring the success of IoT systems that rely on nanosatellites. Their study also discussed future research directions and developments, suggesting that continued advancements in MAC protocols could further enhance the viability of nanosatellites for global IoT connectivity. Lee et al [16] highlighted the importance of high reliability for nanosatellites that must operate autonomously with limited redundancy due to size and weight constraints. To address this, Lee proposed a novel deep learning-based fault management system for detecting and identifying faults in the reaction wheel, a key satellite actuator. The model learns fault patterns by analyzing the residual between measured and estimated attitude data, allowing for autonomous fault detection even without ground station communication. This method was especially relevant for future mega-constellation missions, enhancing the operational autonomy of nanosatellites. Paiva et al [17] introduced CubeSatFI, a fault injection platform designed to enhance the Verification and Validation (V&V) process of CubeSat software by simulating the effects of Single Event Upsets (SEU) caused by space radiation. Given that CubeSats use commercial off-the-shelf (COTS) components, which are highly susceptible to SEU, this platform facilitates fault injection campaigns to emulate bit-flip faults in CubeSat boards through the TAG Test Access Port. The CubeSatFI platform operates in a fully automated mode and was demonstrated using the Environment Data Collection (EDC) payload system, providing insights into the software’s resilience to faults. This tool is intended to support the Brazilian National Institute for Space Research (INPE) for CubeSat constellations. Pancher et al [18] implemented a fault tolerance technique called N-Modular Redundancy and M-Partitions (NMR-MPar) on the MPPA Coolidge many-core processor developed by KALRAY as part of the OVNIPROM1 project. This technique leveraged the multiple cores of the processor to enhance its resilience to radiation-induced errors, making it suitable for use in a nanosatellite On-Board Computer (OBC). The paper outlines how this fault tolerance approach was integrated into the MPPA processor, ensuring that it can effectively manage errors caused by space radiation, thereby improving the reliability of nanosatellite missions. Denby et al [19] identified limitations in the bent-pipe architecture of Earth-observing nanosatellite constellations, where communication is constrained by factors like ground station location, antenna size, and energy availability. To overcome these challenges, they proposed an Orbital Edge Computing (OEC) architecture, which enables local data processing on nanosatellites when downlinking is not feasible. The OEC system organizes satellite constellations into computational pipelines, allowing for parallelized data collection and processing without cross-link coordination. Their results demonstrated that OEC significantly reduces ground infrastructure requirements by 24x and improves edge processing latency by over 617x, compared to the traditional bent-pipe approach.

Table 1 is a summary of the previous work done by space agencies related to the impact of space environments on the integrity of nanosatellite systems, particularly in the context of Single-Event Effects (SEEs):

**Table 1:** Global contributions to SEE studies in nanosatellites.

Agency	Project	Contribution Summary
NASA [20]	CubeSat Radiation Testing	Identified SEE-prone zones in LEO using radiation testing and predictive modeling techniques.
ESA [21]	RadCube	Developed real-time onboard radiation sensors for SEE monitoring and anomaly mapping in small satellites.
JAXA [22]	HORYU-II/IV	Conducted in-orbit tests with COTS components to evaluate susceptibility to SEEs in the LEO environment.
CNSA [23]	Chang’e CubeSat Radiation Exposure	Studied cosmic radiation effects on nanosatellite electronics using dosimeters and space weather models. Noted increased SEE risk during high solar activity.
NASA-JPL [24]	SEE Mitigation in CubeSats	Used hardware redundancy, error-correcting codes (ECC), and shielding. Achieved mitigation with high resource cost.
ISRO [25]	SEU Studies in PSLV-Launched CubeSats	Monitored bit-flips and processor malfunctions using onboard logging. Highlighted need for predictive approaches.
CSA [26]	CUBICS Mission	Researched nanosatellite resilience in high-radiation conditions. Developed radiation-tolerant components and detection systems. Emphasized challenges for small-scale developers.

Despite these advances, most recent approaches either rely on computationally intensive models, focus on post-event analysis, or lack explicit consideration of onboard feasibility and uncertainty estimation. This further motivates the need for efficient, interpretable, and real-time-capable frameworks such as the proposed SEEnet approach.

## Methodology

### Dataset description and feature selection

Our study uses a dataset provided by the Institute of Space Systems at the University of Stuttgart, Germany. The data were collected during the operational phase of a Low Earth Orbit (LEO) satellite mission and were used to develop a predictive model for radiation-induced Single-Event Effects (SEEs), with particular emphasis on memory-related anomalies. SEEs occur when high-energy particles, such as cosmic rays and solar energetic particles, interact with electronic components, potentially disrupting satellite operation and compromising onboard data reliability.

The dataset contains 14,903 samples, each representing a snapshot of the satellite’s operational state. For each sample, the dataset includes geographic longitude, latitude, altitude, a timestamp, and a binary label indicating whether an SEE-related anomaly was observed (“1”) or not (“0”). These labels were derived from onboard telemetry anomaly records and system monitoring logs. A positive label was assigned when abnormal behavior consistent with radiation-induced memory disturbances, such as transient faults or bit flips, was detected within a defined time window. As direct radiation measurements were not available, the labels reflect observed system behavior rather than explicit physical classification of individual SEE subtypes, which aligns with typical nanosatellite operational practices.

Because satellite data are collected along orbital paths, samples from the same orbit pass may be temporally and spatially correlated. To avoid information leakage that could occur with random data splitting, a blocked spatio-temporal partitioning strategy was adopted. Samples were grouped by non-overlapping orbital segments before being divided into training and testing sets. This ensured that data from the same orbit sweep did not appear in both sets, resulting in a more realistic evaluation of model performance. All baseline models and the proposed SEEnet framework were assessed using the same partitioning strategy to allow fair comparison. Before training the models, a feature selection step was performed to improve interpretability and reduce computational complexity. From the available attributes, three spatial features longitude, latitude, and altitude were selected due to their direct relevance in describing the satellite’s orbital position and associated radiation exposure patterns in LEO. These features enable the model to capture spatial trends linked to increased SEE likelihood while remaining suitable for real-time onboard deployment.

Although solar and geomagnetic parameters such as Kp and Ap indices, proton flux, South Atlantic Anomaly indicators, or McIlwain L values are known to strongly influence radiation-related effects, such information was not available in the dataset used in this study. Therefore, the proposed approach should be viewed as a position-based baseline for SEE risk estimation rather than a complete space-weather-driven solution. Nevertheless, a spatial-only model remains practically useful, as orbital position data are continuously available onboard nanosatellites and can provide early, low-cost indications of elevated radiation risk. Our SEEnet framework is primarily designed to be easily extended, and future work will focus on incorporating dynamic space weather and geomagnetic features to further improve prediction accuracy.

#### Handling missing values.

Although the dataset was largely complete, a small subset of samples contained missing values, which, if unaddressed, could negatively impact model accuracy and generalizability. To address this issue, we employed the SimpleImputer method, a commonly used technique in data preprocessing for managing missing data. The imputation strategy was configured to replace missing values with the mean of the corresponding feature.

The imputation process was carried out in two phases:

1. The imputer was first fitted to the training dataset using the fit() method, allowing the computation of mean values for each selected feature (longitude, latitude, and altitude).
2. Subsequently, both training and testing datasets were transformed using the transform() method, ensuring consistent handling of missing values across all subsets of the data.

This imputation approach ensured that the influence of missing values was minimized while preserving the overall statistical properties of the dataset.

#### SimpleImputer for handling missing data.

Accurate handling of missing values within the spatial features was a critical component of during our study for the data preprocessing pipeline, as machine learning algorithms generally require complete datasets to ensure optimal performance. To address this, we employed the SimpleImputer class from the scikit-learn library, a well-established method for managing missing data. This technique enabled the replacement of missing values with statistically meaningful estimates, thereby preserving the integrity of the dataset. Specifically, SimpleImputer was utilized to impute missing entries in the key spatial attributes longitude, latitude, and altitude ensuring consistency and reliability in the dataset used for training and evaluating the SEE prediction model.

Let $X\in {\mathbb{R}}^{n\times m}$ represented our dataset, where *n* is the number of observations and *m* is the number of features. Missing values in *X* are indicated by an indicator matrix $M\in \{0,1{\}}^{n\times m}$, such that:


$$M_{ij}=\left\{ \begin{array}{cc} 1, & \text{if }X_{ij}\text{ is missing}\\ 0, & \text{otherwise} \end{array}\right.$$

The imputation process replaces missing entries in *X* with a chosen statistic *S* (e.g., mean, median). This process can be expressed mathematically as:


$$X_{\text{imputed}}=X\cdot (1-M)+S\cdot M$$

where· denotes element-wise multiplication. This equation ensures that existing (non-missing) values are preserved, while missing entries was replaced with appropriate estimates derived from the non-missing data.

**Algorithm 1** Imputing Missing Values using SimpleImputer

1: **Input:** Dataset *X* with features longitude, latitude, and altitude

2: **Output:** Imputed dataset *X*_imputed_ with no missing values

3: Identify missing values in the spatial features (i.e., NaN entries in longitude, latitude, and altitude)

4: Select imputation strategy: **mean** (average value of each column)

5: Initialize SimpleImputer with strategy set to mean

6: Fit the imputer on the training dataset using fit()

7: Apply the imputer to training and testing data using transform(), replacing missing values with column means

8: Return the cleaned and imputed dataset *X*_imputed_

Following data cleaning and imputation, the dataset was partitioned into training and testing subsets using an 80:20 split [27]. Specifically, 80% of the data was used for training the model, while the remaining 20% was reserved for testing and validation. To ensure reproducibility, a fixed random_state of 42 was set during the partitioning process. This guarantees that the data split remains consistent across different runs of the code, allowing for reliable and comparable evaluation of model performance. During the process, random_state parameter acted as a seed for the random number generator used in processes such as data shuffling and splitting. By setting random_state = 42, we ensured that the same train-test division was reproduced every time the code is executed under identical conditions.

### Voting classifier

To enhance the predictive performance of our SEE (Single-Event Effect) classification model, we employed a voting classifier based on ensemble learning principles. Ensemble methods combine the outputs of multiple base learners to improve overall model accuracy and robustness. In this study, the voting classifier integrated several individual classifier denoted as $m_{1},m_{2},\ldots ,m_{n}$ each trained on the spatial features (longitude, latitude, and altitude) associated with satellite telemetry data.

Two primary aggregation strategies were considered: *hard voting* and *soft voting*. In the hard voting approach, each base model *m*_*i*_ generates a discrete class label prediction *y*_*i*_ for a given input *x*, and the final class label *y*_final_ is determined by majority vote:


$$y_{\text{final}}=\arg\max_{y}\left\{\sum_{i=1}^{n}1\left[m_{i}(x)=y\right]\right\}$$

where 1[·] is the indicator function that returns 1 if the condition is true and 0 otherwise.

In contrast, the soft voting strategy relied on the probabilistic outputs of each base model. Specifically, each model *m*_*i*_ predicts a probability distribution $P_{i}(y|x)$ over the class labels. The final prediction is then obtained by averaging these probabilities across all models and selecting the class with the highest average probability:


$$y_{\text{final}}=\arg\max_{y}\left\{\frac{1}{n}\sum_{i=1}^{n}P_{i}(y|x)\right\}$$

By aggregating predictions from multiple diverse classifiers, the voting mechanism reduced the risk of overfitting and enhances the generalization capability of the ensemble. This methodology was particularly advantageous in our study, where the occurrence of SEEs is influenced by complex spatial patterns. The ensemble approach effectively captured such patterns, outperforming individual classifiers and contributing to a more robust predictive framework. In this study, the soft voting strategy was adopted for the final SEEnet implementation, as it allows the ensemble to leverage the confidence information provided by individual classifiers.

### Proposed algorithm

#### Overview of a nano-satellite system.

**Fig 1:**
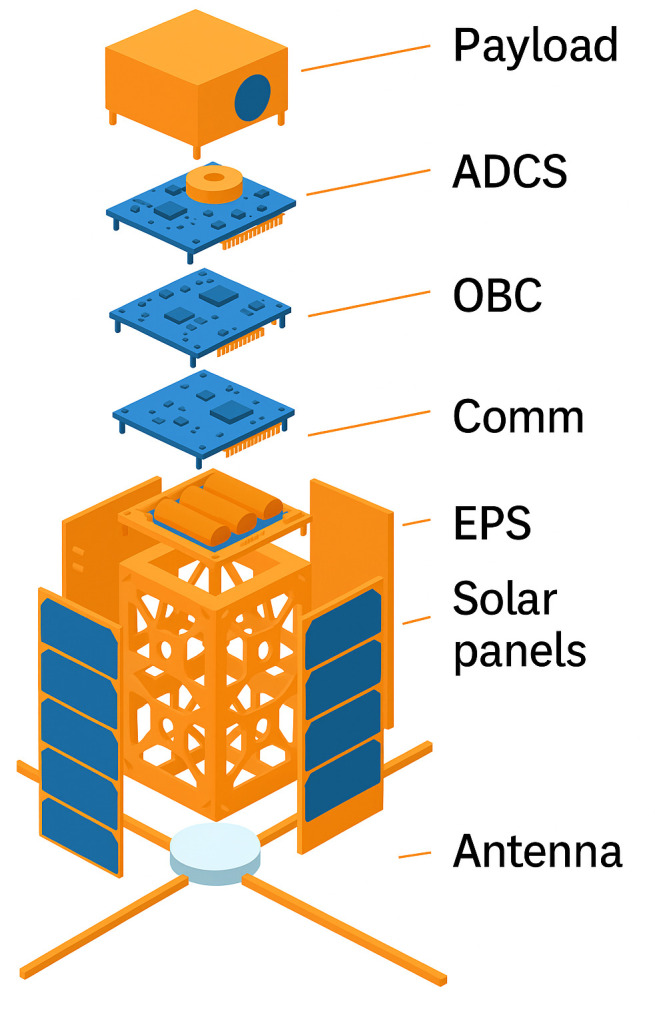
Structural analysis of nanosatellite.

**Fig 2:**
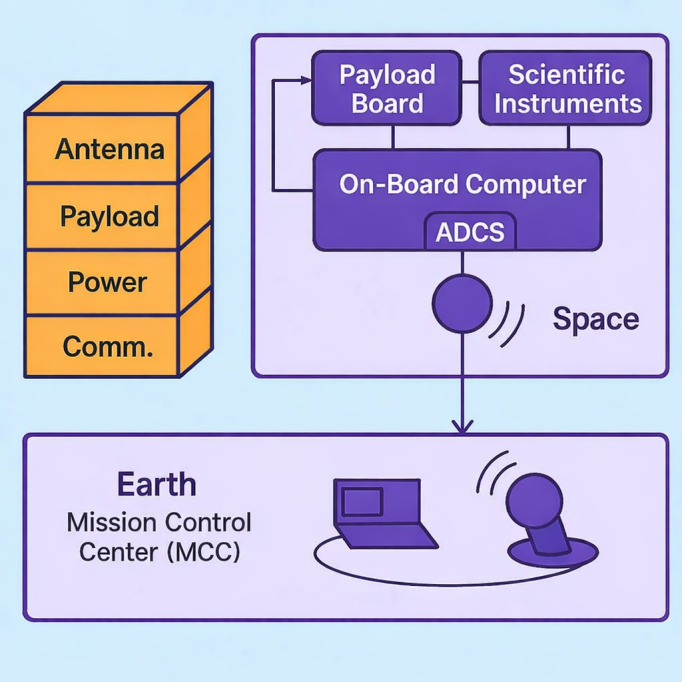
Overview of how nano-satellite system works.

Single Event Effects pose a significant threat to the reliability of nanosatellite systems, particularly with respect to data integrity. Memory components such as RAM, EEPROM, and flash storage are especially vulnerable to Single Event Upsets (SEUs) displayed in Fig 1, which can result in bit flips and data corruption, affecting telemetry, command execution, and scientific data retention. Processors (CPUs and FPUs) may experience faulty computations or software malfunctions due to SEEs, while communication subsystems can suffer disruptions in transceivers or modulators, leading to corrupted data transmission between the satellite and ground stations, as visible in Fig 2. Data storage devices are at risk of permanent corruption, and sensors may generate inaccurate readings, compromising mission outcomes. The On-Board Computer (OBC), which governs system coordination and data processing, is particularly susceptible, where a single fault can impact the entire satellite’s operation. Though power systems are less directly related to data integrity, SEE-induced failures can disrupt power distribution, indirectly affecting data-critical subsystems.

**Fig 3:**
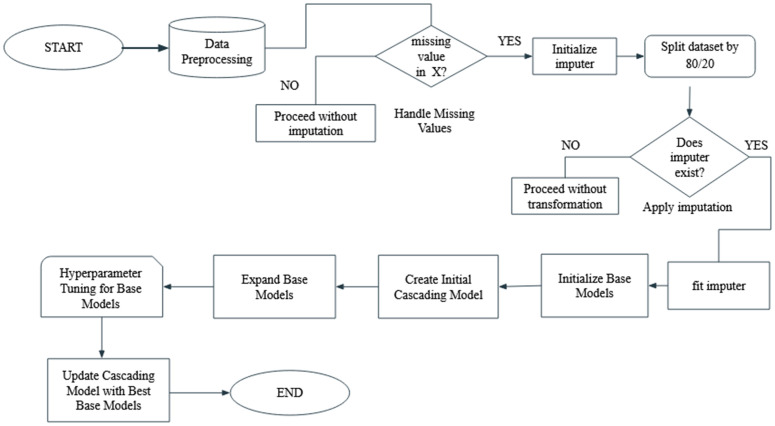
Flowchart of our proposed SEEnet.

The system architecture shown in Fig 3 was deployed to understand the internal communication flow and subsystem interactions within a typical CubeSat platform. The configuration provides a practical reference model for analyzing the operational context in which SEEs are likely to occur. Specifically, the system highlights how the On-Board Computer (OBC) functions as the central node for managing communications between the satellite and the Mission Control Center (MCC), as well as with internal subsystems such as the Attitude Determination and Control System (ADCS). This communication structure is essential for identifying how SEE events may propagate across subsystems and affect satellite behavior. Moreover, the serial link between the OBC and the FPGA-based payload board, alongside high-speed connections with scientific instruments, reflects realistic data pathways through which radiation-induced anomalies could be monitored or logged. The modular CubeSat design, with stacked 10 cm cube units, further demonstrates the physical constraints and integration challenges inherent to implementing radiation prediction mechanisms.

#### Our SEEnet framework.

**Fig 4:**
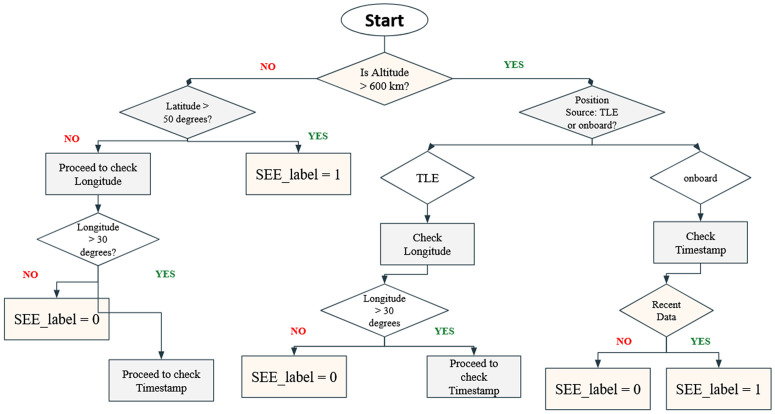
Decision tree flowchart for SEE_label prediction based on satellite telemetry parameters.

The methodology adopted in our study followed a structured and iterative approach to develop and optimize the proposed *SEEnet* algorithm for the prediction of single-event effects (SEEs) in nanosatellites shown in Fig 4. Initially, two base learners were constructed using Decision Tree classifiers with maximum depths of 3 and 5, respectively. These models were integrated into an ensemble framework using a soft voting strategy to form the initial version of our SEEnet [28]. The ensemble was trained on the designated training dataset and subsequently evaluated on a separate test dataset, achieving an initial classification accuracy of 77%. To enhance the model’s performance, the ensemble was expanded by incorporating two additional Decision Tree classifiers with maximum depths of 4 and 6. The inclusion of these models introduced additional diversity within the ensemble, and retraining the updated configuration yielded improved predictive accuracy. Recognizing the opportunity for further refinement, hyperparameter optimization was performed using the GridSearchCV utility from the scikit-learn library. This process involved an exhaustive search over a defined parameter grid to identify the optimal values of max_depth for each base learner. The best-performing models from this tuning procedure were then integrated into SEEnet. Upon retraining, the optimized ensemble demonstrated enhanced predictive performance, underscoring the effectiveness of both model diversification and hyperparameter tuning in improving classification outcomes for SEE prediction.

**Fig 5:**
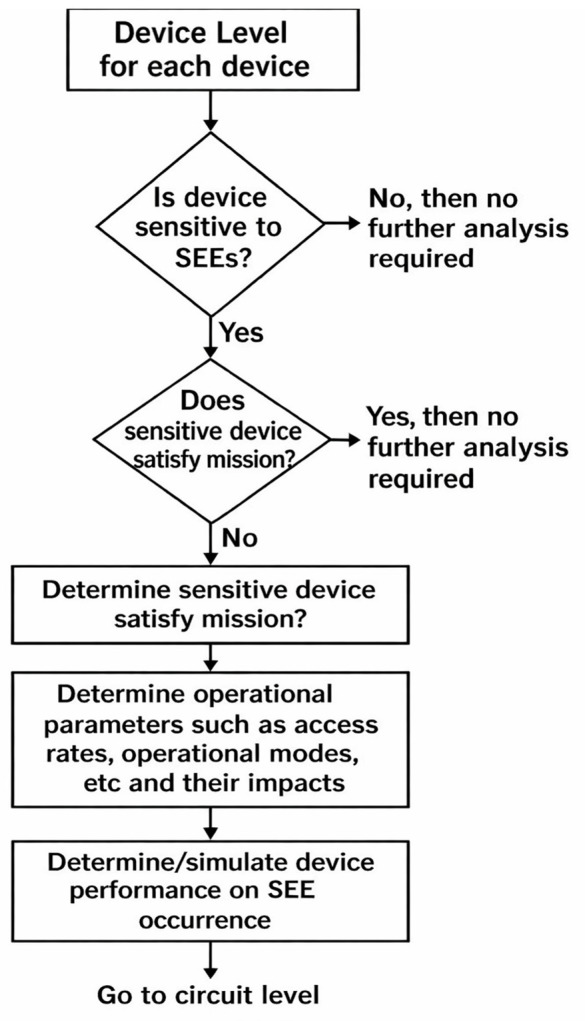
Device level SEE propagation methodology.

A domain-specific, rule-based decision tree was developed to perform preliminary labeling of Single Event Effects (SEEs) using key orbital and positional attributes. The flowchart, illustrated in Fig 5, initiates the classification process by assessing the altitude of the nanosatellite. If the altitude is below or equal to 600 km, the model evaluates the geospatial context by examining the latitude.

Specifically, if the latitude exceeds 50°, the location is considered high-risk due to increased exposure to charged particles in polar regions, and the sample is labeled as SEE-prone (SEE_label = 1). If this condition is not satisfied, the model proceeds to assess the longitude. For longitudes greater than 30°, temporal attributes such as timestamp data are further analyzed for potential correlations with space weather activity. Otherwise, the instance is classified as non-SEE (SEE_label = 0). Conversely, if the satellite’s altitude exceeds 600 km typically characteristic of Medium Earth Orbit (MEO) or Low Earth Orbit (LEO) in high-radiation belts, the model distinguishes between Two-Line Element (TLE) data and onboard position readings. For TLE-based positional data, the longitude is again used as an indicator of spatial vulnerability, followed by timestamp analysis. In the case of onboard sensor data, the recency of the timestamp is considered crucial; recent readings, especially those aligned with known solar activity, are labeled as SEE events. The decision tree serves as a transparent baseline method to support our framework. It also provides robust fallback labeling in scenarios where ground truth is sparse or unavailable. The hybridization of this rule-based approach with ensemble learning strengthens the overall predictive framework for SEEs in nanosatellites.

Beyond numerical performance gains, the improved behavior of SEEnet can be attributed to the complementary strengths of ensemble learning. Decision-tree-based models are sensitive to data variations, and combining multiple learners helps reduce this sensitivity by averaging out individual model errors. In particular, ensemble learning mitigates variance by aggregating diverse decision boundaries while improving generalization compared to a single classifier. This allows SEEnet to better capture heterogeneous spatial patterns associated with radiation exposure across different orbital regions. In addition, the bootstrap-based uncertainty estimates provide information beyond point predictions. Although uncertainty is not directly used for decision gating in the current implementation, it offers a valuable operational signal. For example, predictions associated with higher uncertainty could trigger precautionary actions such as memory scrubbing, increased error checking, or temporary mode switching, whereas low-uncertainty predictions may allow normal operation to continue. Such uncertainty-aware strategies can support more informed and adaptive fault mitigation in future nanosatellite missions.

**Algorithm 2** Imputing Missing Values using SimpleImputer

1: **Input:** Dataset with columns: Longitude (Lon), Latitude (Lat), Altitude (Alt), Timestamp, SEE Label (SEElabel)

2: **Output:** Predictions of SEE events

3: Identify missing values in the spatial features (i.e., NaN entries in longitude, latitude, and altitude)

4: **Data Preparation:**

5:  X←[Lon,Lat,Alt], y←SEElabel

6: **Handle Missing Values:**

7: **if** any missing values in *X* **then**

8:  Initialize SimpleImputer: $\text{imputer}\leftarrow \text{SimpleImputer}(\text{strategy}{=}^{\prime }mean^{\prime })$

9: **else**

10: Proceed without imputation

11: **end if**

12: **Split the Dataset:** Train-test split of *X* and *y*

13: **Apply Imputation:**

14: **if** imputer is defined **then**

15:  imputer.fit(*X*_train_)

16:  $X_{\text{train}}\leftarrow \text{imputer.transform}(X_{\text{train}})$

17:  $X_{\text{test}}\leftarrow \text{imputer.transform}(X_{\text{test}})$

18: **else**

19:  Proceed without transformation

20: **end if**

21: **Initialize Base Models:**

22:  Define base models: Decision Trees with varying max_depth values

23: **Create Initial Ensemble Model:**

24: SEEnet ← VotingClassifier(estimators = [(’model1’, model1), (’model2’, model2)], voting=’soft’)

25: **Train and Evaluate Initial Model:**

26: Fit SEEnet on training data and evaluate performance

27: **Expand Base Models:**

28:  Add additional Decision Tree classifiers (e.g., max_depth = 4, 6)

29: **Hyperparameter Tuning for Base Models:**

30:  Use GridSearchCV to identify optimal max_depth values

31: **Update Ensemble with Best Base Models:**

32: **if** Grid search complete **then**

33:  Replace base models with best estimators from grid search

34: **else**

35:  Use original base models

36: **end if**

37: **Hyperparameter Tuning for Ensemble Model:**

38:  Define parameter grid: param_grid_ensemble = {’voting’: [’soft’, ’hard’]}

39: Apply GridSearchCV on SEEnet with defined grid

40: **Update Ensemble with Best Voting Strategy:**

41: **if** Ensemble grid search complete **then**

42:  SEEnet ← best estimator from ensemble grid search

43: **else**

44:  Retain original ensemble configuration

45: **end if**

46: **return** Predictions from optimized SEEnet

## Experiment and performance analysis

### SEE propagation analysis

Single Event Effect (SEE) Propagation refers to the process by which the impact of a localized SEE, such as a single event upset (SEU), single event transient (SET), single event latch-up (SEL) or single event burnout (SEB), extends beyond the initial site of occurrence within a microelectronic component to influence higher levels of a system’s architecture. This phenomenon encompasses the analysis of how an SEE-induced fault in a specific device can affect the behavior of the associated circuitry, propagate through interconnected subsystems, and ultimately compromise the functionality of the entire system or spacecraft. SEE propagation is of particular concern in spaceborne and high-reliability systems, where even a transient or localized fault may result in significant operational anomalies if left unmitigated. For example, a single event transient occurring in a timing-critical digital circuit may lead to incorrect data processing, which in turn may cause erroneous control commands to be issued at the system level. The propagation of such effects underscores the necessity of system-level modeling, error containment strategies, and fault-tolerant design methodologies in environments susceptible to ionizing radiation.

In many ways, SEE propagation shares similarities with both traditional circuit simulation and Failure Modes and Effects Analysis (FMEA). In both approaches, the primary objective is to understand the consequences that a localized disturbance, whether an error or a failure, has on the overall performance and reliability of a device, circuit, or system. SEE propagation, however, extends this analysis to account for a wider spectrum of radiation-induced phenomena, such as single event transients, latch-ups, and burnouts, each with unique temporal and spatial characteristics. The aim is to assess how a single event originating at the microelectronic level may evolve and impact system-level behavior, potentially compromising mission-critical functions.

To systematically assess and predict the susceptibility of nanosatellite components to Single Event Effects (SEE), a device-level flowchart methodology was adopted, as illustrated in Fig 5. The process begins by evaluating each onboard device to determine whether it is sensitive to SEE-induced disruptions. If a device is found to be insensitive, no further analysis is required. However, if sensitivity is confirmed, the next step is to assess whether the device can still fulfill its intended mission functions despite potential SEE occurrences. Devices that fail this criterion are flagged for detailed investigation. The methodology then proceeds to identify specific sensitive areas within the device, such as memory cells or control registers, that are most vulnerable to radiation-induced faults. Following this, key operational parameters including access RAM modes, device functional states, and system clock characteristics are analyzed to understand their impact on SEE susceptibility. Finally, the device’s performance under SEE conditions is either determined experimentally or simulated, enabling a comprehensive understanding of how SEEs may affect system reliability. This bottom-up approach allowed our study to trace the propagation of faults from the device to the circuit level, ultimately contributing to a more accurate and data-driven model for predicting SEE occurrences in nanosatellite systems.

### Experimental analysis

**Fig 6:**
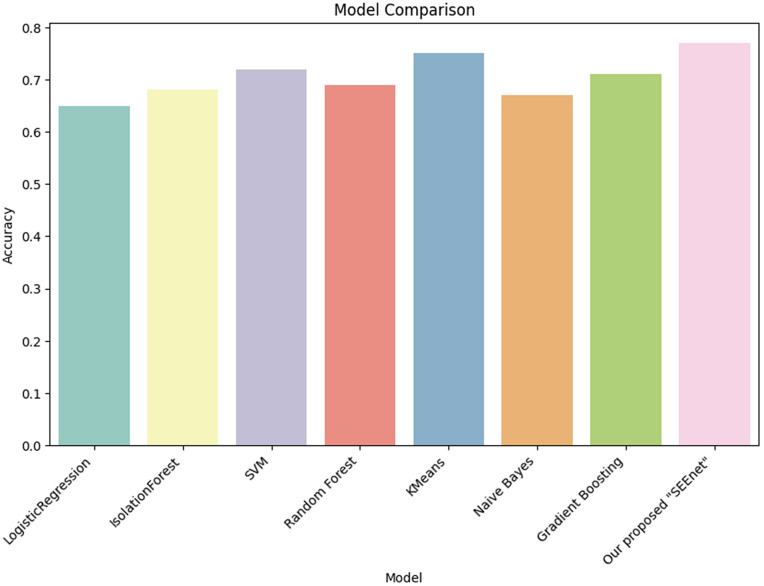
Comparison of our algorithm via various ML models.

To evaluate the effectiveness of our proposed SEEnet algorithm for predicting single-event effects (SEEs) in nanosatellites, we conducted a comparative performance analysis against several established machine learning models shown in Table 2. Each model was trained and tested on the same dataset, ensuring consistency in evaluation. Because SEE events occur significantly less frequently than non-SEE events, the dataset exhibits class imbalance. For this reason, overall classification accuracy is reported for completeness but is not interpreted in isolation. Greater emphasis is placed on class-wise precision, recall, F1-score, and ROC-AUC, which provide a more reliable assessment of model performance on the minority SEE class. SEEnet achieved the highest accuracy at 77%, outperforming all other algorithms. Fig 6 demonstrates its superior ability to correctly predict SEEs in nanosatellites. Gradient Boosting followed closely with an accuracy of 75%, indicating its robustness in handling the dataset. Random Forest achieved an accuracy of 72%, showing strong performance but less effective than SEEnet. SVM, Naive Bayes, Logistic Regression, and Isolation Forest had lower accuracies of 70%, 68%, 65%, and 63% respectively. In case of Precision, Recall, and F1-Score SEEnet showed the highest precision at 0.78, indicating its effectiveness in minimizing false positive predictions. In terms of recall, SEEnet achieved 0.75, highlighting its strong performance in correctly identifying actual SEEs. The F1-Score of SEEnet was 0.76, the highest among all algorithms, reflecting its balanced performance between precision and recall. SEEnet also excelled in the ROC-AUC metric with a score of 0.82, underscoring its robust capability to distinguish between SEE and non-SEE instances.When placed in comparison to other model, SEEnet’s unique strengths and systematic enhancements offer an insightful perspective on how it performs in relation to more traditional and advanced models.

**Table 2:** Evaluating the performance of our algorithms with different models.

Algorithm	Acc.	Pre.	Recall	F1	ROC-AUC
Logistic Regression	65%	0.67	0.64	0.65	0.70
Support Vector Machine	69%	0.71	0.68	0.69	0.75
Random Forest	72%	0.73	0.70	0.71	0.78
Isolation Forest	63%	0.65	0.60	0.62	0.68
Naive Bayes	68%	0.69	0.66	0.67	0.72
Gradient Boosting	75%	0.76	0.74	0.75	0.80
**Our proposed SEEnet**	**77%**	**0.78**	**0.75**	**0.76**	**0.82**

**Fig 7:**
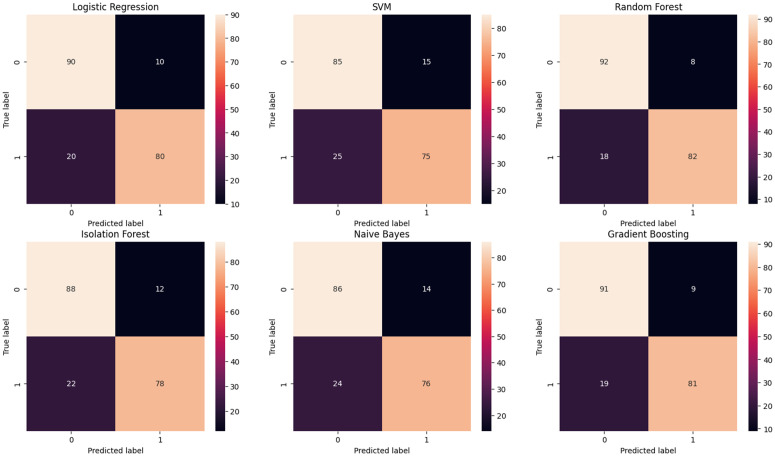
Confusion Matrix of different ML models.

**Fig 8:**
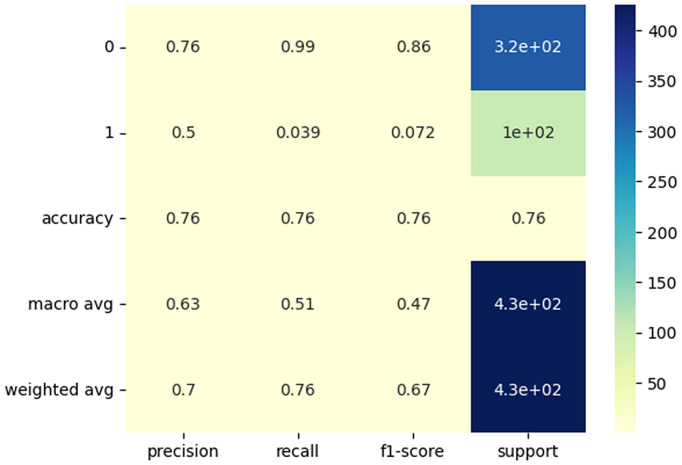
Heatmap representation of SEEnet prediction outcomes on the test dataset.

Fig 7 represents the confusion matrix classification performance of different machine learning and a binary classification task illustrating the trade-off between false positive and false negative predictions across different machine learning models and Fig 8, where the two classes are labeled as 0 and 1. The model performs well in predicting class 0, with a precision of 76%, recall of 99%, and an F1-score of 86%. This indicates that the model is highly accurate in identifying class 0 instances, which also make up the majority of the dataset with 320 instances. However, the performance for class 1 is significantly poorer, with a precision of 50%, recall of only 3.9%, and an F1-score of 7.2%, suggesting that the model struggles to correctly identify and predict class 1 instances. The overall accuracy of the model is 76%, meaning it correctly predicts 76% of all instances. The macro averages (which treat both classes equally) show a substantial drop in recall and F1-score, further highlighting the model’s imbalance in performance. The weighted averages, which account for class distribution, show better precision (70%) and recall (76%), but the F1-score of 67% reflects that the model’s overall performance is primarily driven by the correct classification of the majority class (class 0). This imbalance suggests a need for improvement in handling the minority class (class 1), perhaps through methods like rebalancing the dataset or employing more advanced classification techniques. Class imbalance was addressed at the evaluation stage by emphasizing class-wise performance metrics rather than relying solely on overall accuracy.

From an operational perspective, the observed imbalance between false positive alerts and missed SEE detections represents an important trade-off rather than a purely methodological limitation. In nanosatellite missions, missed SEE events (false negatives) may lead to data corruption or system instability, whereas false positives typically trigger precautionary mitigation actions such as memory scrubbing or temporary mode switching. As illustrated by the confusion matrix and heatmap representations, the current model configuration prioritizes overall stability under class imbalance, with performance dominated by the majority class. Threshold tuning and mission-specific decision strategies may be explored in future work to adjust this balance toward higher SEE recall when operational requirements demand greater sensitivity.

**Fig 9:**
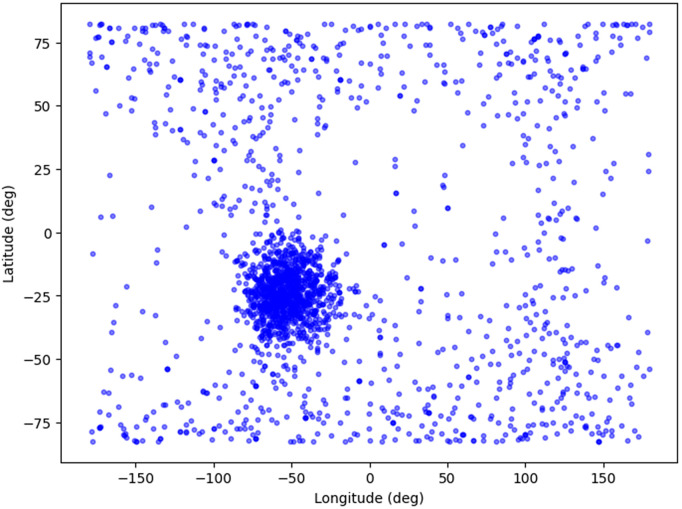
Geo-spatial distribution of SEE events.

**Fig 10:**
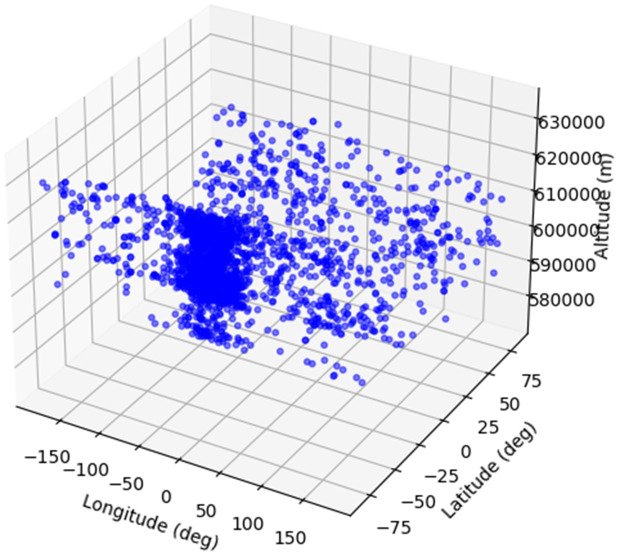
3D plot Geo-spatial distribution of SEE events.

3D scatter plot illustrated in Figs 9 and 10 shows the geospatial distribution of Single Event Effects (SEE) events, which are disruptions in satellite electronics caused by cosmic radiation. The plot maps SEE occurrences across three dimensions: longitude, latitude, and altitude. Longitude, shown on the x-axis, spans from −150° to 150°, representing the east-west distribution, while latitude on the y-axis covers a range from −75° to 75°, indicating the north-south spread. The z-axis depicts the altitude in meters, ranging from 580,000–630,000 meters, capturing the vertical distribution at high altitudes, likely corresponding to satellite orbits. The blue points clustered densely in certain regions suggest that SEE events are more frequent at specific geographic locations and altitudes. This visualization offers valuable insights into the spatial patterns of SEE occurrences, aiding in better understanding and potential mitigation strategies for satellite operators.

**Fig 11:**
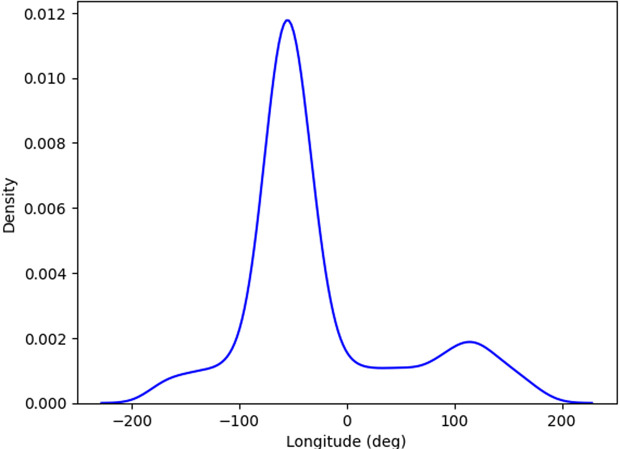
Kernel Density Estimate (KDE) plot.

KDE plot (Kernel Density Estimation) shown in Fig 11 represents the density of data points across different longitude values. The x-axis covers longitudes ranging from approximately −200° to 200°, while the y-axis represents the density of occurrences at each longitude. The peak of the curve is around −50° longitude, indicating that most of the data points are concentrated in this region, as shown by the highest density value (0.012). The curve is smooth, which is typical for KDE plots, as they estimate the probability density function of the data without assuming a specific distribution. The sharp rise and fall around the peak suggest that a significant portion of the data is clustered near −50°, with much lower densities at longitudes closer to −200° and 200°. This KDE plot is useful for identifying where the bulk of the data lies in terms of longitude and for visually identifying regions with sparse data points.

**Fig 12:**
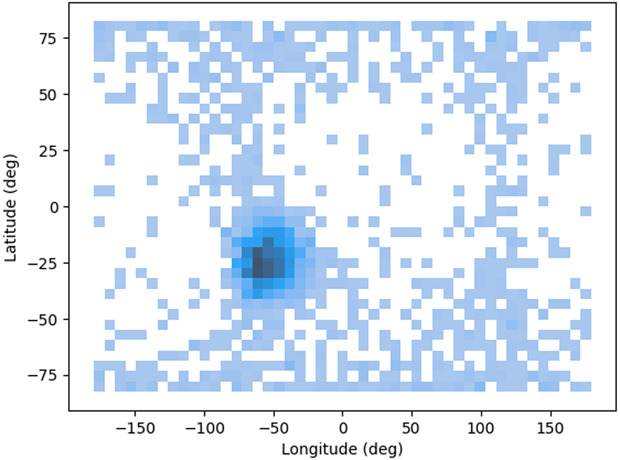
Hexbin plot.

Hexbin plot displayed in Fig 12 illustrates the relationship between Longitude and Latitude, offering insights into the geographical distribution of data points. The x-axis represents longitude, spanning from approximately −150° to 150°, which signifies the east-west positioning on the Earth’s surface. Meanwhile, the y-axis corresponds to latitude, covering a range from −75° to 75°, indicating the north-south positioning. The plot uses hexagonal bins to display the density of the data points, with varying color intensities representing different densities. Darker hexagons show areas with a higher concentration of data points, particularly focused around the coordinates of approximately −25° latitude and −50° longitude. Lighter hexagons represent regions with fewer data points. This type of visualization is useful in understanding how data is spatially distributed, and in this case, it highlights a significant clustering in one central region, likely indicating a geographic hotspot or area of particular interest.

**Fig 13:**
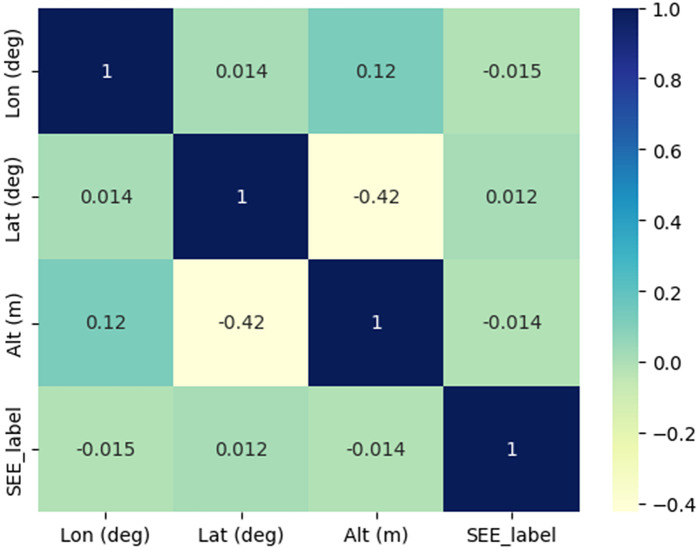
Correlation matrix between different parameters.

Correlation matrix in Fig 13 provides an overview of the relationships between four variables: Longitude (Lon), Latitude (Lat), Altitude (Alt), and a categorical label referred to as SEE_label. As expected, each variable is perfectly correlated with itself, indicated by the values of 1 along the diagonal. Longitude shows a slight positive correlation with Altitude (0.12), suggesting a weak association between higher longitudes and increased altitude, while it exhibits a minor negative correlation (−0.015) with the SEE_label, implying only a very small inverse relationship. Latitude, on the other hand, has a moderate negative correlation (−0.42) with Altitude, indicating that as the latitude increases (moving towards the poles), the altitude generally decreases. Lastly, the SEE_label variable exhibits very weak correlations with all other variables, suggesting that it is not significantly influenced by them. Overall, the matrix helped to identify a few notable, though relatively weak, relationships between these geographic and categorical variables.

**Fig 14:**
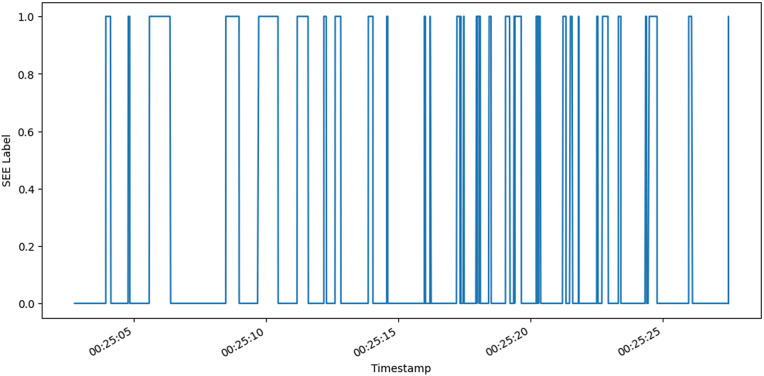
Time-series plot over a period of time.

Time Series Plot of SEE Label illustrates the changes in SEE occurrences over a period of time shown in Fig 14. The x-axis represents the timestamp, while the y-axis denotes the SEE label, where ‘1‘ signifies the presence of an SEE and ‘0‘ indicates its absence. The plot shows a series of alternating spikes between 0 and 1, indicating that SEEs occur intermittently during the observed time period. The frequent and rapid transitions suggest that the system being monitored experiences repeated SEEs, which may require mitigation strategies, especially in radiation-prone environments like space. This plot was useful for analyzing how often SEEs occur and for identifying patterns in their distribution over time. For instance, clustering of ‘1‘s can highlight periods of higher SEE activity, which might be linked to external factors like solar radiation or other environmental influences. Understanding these patterns can be crucial in designing fault-tolerant systems for nanosatellites or other space-bound technologies.

To ensure fair comparison and reproducibility, all base classifiers used in this study were trained and evaluated under the same data partitioning and validation setup. The proposed SEEnet framework consists of decision tree (DT), random forest (RF), and gradient boosting (GB) classifiers. For the decision tree model, the maximum tree depth was varied within a predefined range to control model complexity. Hyperparameter tuning was performed using grid search within reasonable ranges selected based on prior studies and empirical performance. All models shared the same blocked spatio-temporal training and testing split, as described in the dataset section, to prevent information leakage due to orbital autocorrelation. A fixed random seed was used across all experiments to ensure result reproducibility.

### Validation of our algorithm

The growing use of nanosatellites in Low Earth Orbit (LEO) has increased the demand for reliable and adaptive methods to address radiation-induced anomalies that threaten onboard data integrity. Our proposed SEEnet framework is validated not only in terms of predictive performance but also with respect to robustness, interpretability, and practical feasibility for real-world nanosatellite missions. Unlike approaches that focus on isolated fault mechanisms or offline analysis, SEEnet is designed as a general-purpose predictor of radiation-induced SEE occurrence using readily available onboard information. By leveraging an ensemble of decision-tree-based models and spatial telemetry features, SEEnet provides a flexible and lightweight solution capable of operating under dynamic orbital conditions. The comparative results summarized in Table 3 demonstrate that SEEnet consistently outperforms individual baseline models, highlighting the benefits of ensemble learning for improved generalization.

**Table 3:** Comparison of SEEnet with existing SEE prediction models.

Aspect	Our proposed SEEnet	J. Chen et.al [29]	A. Gupta et al [30]	C. Xu et al [31]
**Phenomenon Modeled**	Multiple SEEs in nanosatellites (SEU, SEL, SEB, etc.)	SRAM SEUs during Solar Particle Events (SPEs)	SEUs in memory devices	SET pulse currents in circuits
**Machine Learning Approach**	VotingClassifier with optimized Decision Trees	Basic offline ML + online learning for SEUs	Positional data only for SEU prediction	Neural network for SET pulse modeling
**Data Input**	Diverse nanosatellite data (mission/ environmental factors)	SRAM data as particle detector	Positional data only	LET and bias voltage from simulations
**Accuracy**	77% accuracy for various SEEs	Predicts SEUs 1 hour in advance, specific to SRAM	Accuracy unspecified, likely lower	Accuracy unspecified, simulation-based
**Scalability and Cost**	Highly scalable, cost-effective across CubeSat missions	Limited by SRAM dependency, higher cost	Scalable but simplistic, positional data limits effectiveness	Not scalable beyond circuit simulations
**Impact on Space Missions**	Real-time, multi-SEE protection for nanosatellites	Focused on SRAM SEU mitigation, limited scope	Enhances memory reliability but too narrow for overall SEE risk	Circuit-specific insights, not relevant to full missions

To further assess the stability of the reported results, bootstrap resampling was applied during evaluation to estimate uncertainty in model performance metrics. Multiple bootstrap samples were drawn from the test set, and evaluation metrics were recomputed for each resample. The resulting distributions were used to derive confidence intervals for key performance measures such as accuracy and ROC-AUC. These uncertainty estimates are used for descriptive and interpretative purposes only, providing insight into the variability and robustness of the model’s performance. In the current implementation, uncertainty estimates do not influence the classification decision process, and final predictions are obtained using a fixed probability threshold applied to the ensemble output.

In addition to predictive reliability, practical feasibility is a critical aspect of validating SEEnet for nanosatellite applications. Although this study does not include direct hardware-level implementation or benchmarking, the suitability of SEEnet for real-time onboard deployment can be evaluated based on its computational structure. Each decision tree performs inference through a limited number of simple threshold comparisons along a root-to-leaf path, resulting in low computational overhead. For the ensemble, the total inference cost scales linearly with the number of base models and their respective tree depths, making execution efficient even on resource-constrained embedded processors. The memory footprint of SEEnet is modest, as it primarily consists of storing tree parameters such as feature indices, split thresholds, and leaf values. This compact representation is well aligned with the memory constraints of typical nanosatellite onboard computers. During operation, SEEnet performs brief inference computations, and therefore its expected power consumption remains low compared to more complex learning architectures such as deep neural networks. It should be noted that bootstrap-based uncertainty estimation is performed offline during evaluation and does not introduce additional computational overhead during onboard inference. While precise measurements of inference time, memory usage, and power consumption require benchmarking on flight-representative hardware, the complexity-based analysis presented here supports the practical feasibility of deploying SEEnet for real-time SEE risk assessment. Direct hardware validation is identified as an important direction for future work.

## Conclusion

Our study successfully demonstrated the effectiveness of SEEnet, a novel machine learning algorithm designed to predict Single-Event Effects (SEEs) in nanosatellites. Through a comparative analysis, SEEnet consistently outperformed other machine learning models, achieving the highest accuracy of 77%, along with superior precision, recall, and F1-Score. The algorithm’s ability to minimize false positives while accurately detecting SEEs makes it an optimal solution for real-time SEE prediction. Furthermore, SEEnet’s ROC-AUC score of 0.82 reinforces its strong discriminatory capability, indicating that it can reliably distinguish between SEE and non-SEE events. These results confirm the viability of SEEnet for improving the data integrity and operational reliability of nanosatellites in the unpredictable environment of space. The central contribution of our research is the development of SEEnet, a novel machine learning algorithm specifically designed to predict SEEs in real-time. SEEnet leverages advanced ensemble learning techniques, combining multiple models to achieve superior prediction accuracy. By outperforming traditional machine learning models such as Gradient Boosting, Random Forest, and Support Vector Machines (SVM), SEEnet demonstrates its effectiveness in detecting SEEs with an accuracy of 77%, making it one of the most reliable solutions for SEE prediction in nanosatellites.

Another key contribution of the study lies in its ability to significantly enhance data integrity in nanosatellites. SEEs, which can corrupt onboard data by causing bit flips or other memory errors, pose a major risk to mission-critical operations. SEEnet addresses this issue by accurately predicting the occurrence of SEEs, enabling nanosatellite systems to take preemptive actions to protect data integrity. With its high precision and recall, SEEnet effectively minimizes false positives while accurately identifying actual SEEs, providing a balanced and robust solution to prevent data corruption in space missions. Furthermore, the study contributes to the autonomous operation of nanosatellites by integrating real-time SEE prediction into the satellite’s On-Board Computer (OBC). SEEnet’s ability to function autonomously, even in the absence of continuous communication with ground stations, enhances the reliability of nanosatellite operations, particularly in environments where ground communication is limited. This autonomous functionality is critical for nanosatellites operating in Low Earth Orbit (LEO), where communication windows with ground stations are brief and infrequent.

Additionally, this research provides a foundation for future advancements in SEE prediction and nanosatellite resilience. The framework developed in this thesis can be expanded to incorporate real-time space weather data, further improving the model’s predictive accuracy by dynamically adjusting to changing space conditions. The research also suggests potential applications for SEEnet in multi-satellite constellation [32], where nanosatellites can share SEE data to collectively improve prediction accuracy across a network. Moreover, this study proposes the integration of autonomous mitigation strategies, such as system reconfiguration or data backups, that can be automatically triggered by SEEnet when a SEE is predicted, ensuring minimal disruption to satellite operations. It also offers practical advancements that will benefit future nanosatellite missions by ensuring data integrity, supporting autonomous operations, and laying the groundwork for more complex satellite systems in space exploration.

### Future prospects of our work

The proposed SEEnet framework opens several promising directions for future research and development. One important extension involves incorporating real-time space weather information, such as solar activity, geomagnetic indices, and cosmic radiation levels. Integrating these dynamic environmental factors would allow SEEnet to respond more effectively to changing radiation conditions and further improve the accuracy and reliability of SEE risk prediction in operational space environments. Another valuable direction is the expansion of SEEnet to multi-satellite constellation scenarios. In such settings, nanosatellites operating within a constellation could share SEE-related observations and environmental information, enabling collaborative learning across multiple platforms. This cooperative prediction strategy could enhance situational awareness and improve overall system resilience, particularly for Earth observation and communication missions involving distributed satellite networks. Future work will also focus on extending the validation of SEEnet using additional publicly available datasets related to radiation effects and anomaly detection in space systems. While many existing datasets emphasize space weather indices, simulated radiation environments, or component-level irradiation experiments, combining these datasets with operational telemetry-based data will enable broader evaluation across diverse mission profiles. Such comparative studies will help assess generalizability and support the development of more comprehensive, data-driven SEE prediction frameworks. Finally, integrating autonomous mitigation mechanisms with SEEnet represents a key opportunity to enhance nanosatellite resilience. By coupling SEE prediction with automated preventive actions such as system reconfiguration, enhanced error correction, or proactive data protection, future implementations could enable nanosatellites to respond independently to radiation threats. Together, these advancements would allow SEEnet to evolve into a more adaptive and scalable framework, supporting reliable operation across increasingly complex and long-duration space missions.
